# Physico-Chemical and Antiadhesive Properties of Poly(Lactic Acid)/Grapevine Cane Extract Films against Food Pathogenic Microorganisms

**DOI:** 10.3390/polym12122967

**Published:** 2020-12-12

**Authors:** Edaena Pamela Díaz-Galindo, Aleksandra Nesic, Gustavo Cabrera-Barjas, Octavio Dublan-García, Rosa Isela Ventura-Aguilar, Francisco Javier Vázquez-Armenta, Saúl Aguilar-Montes de Oca, Claudia Mardones, Jesús Fernando Ayala-Zavala

**Affiliations:** 1 Facultad de Química, Universidad Autónoma del Estado de México, km 115 Car, Toluca-Ixtlahuaca, El Cerillo Piedras Blancas, Toluca 50295, Mexico; pam.dg12@hotmail.com (E.P.D.-G.); odublang@uaemex.mx (O.D.-G.); 2 Unidad de Desarrollo Tecnológico (UDT), Universidad de Concepción, Avda. Cordillera No. 2634, Parque Industrial Coronel, Coronel 4191996, Chile; a.nesic@udt.cl; 3 Department of Chemical Dynamics and Permanent Education, Vinca Institute of Nuclear Sciences—National Institute of the Republic of Serbia, University of Belgrade, Mike Petrovica-Alasa 12-14, 11000 Belgrade, Serbia; 4 CONACYT-Centro de Desarrollo de Productos Bióticos, Instituto Politécnico Nacional, Carretera Yautepec-Jojutla, San Isidro, Yautepec 62731, Morelos, Mexico; riventuraag@conacyt.mx; 5 Centro de Investigación en Alimentación y Desarrollo, A. C. Carretera Gustavo Enrique Astiazarán Rosas No. 46, Col. La Victoria, Hermosillo 83304, Sonora, Mexico; javiervazarmenta@gmail.com; 6 Centro de Investigación y Estudios Avanzados en Salud Animal, Facultad de Medicina Veterinaria y Zootecnia (CIESA-FMVZ-UAEM), Autopista Toluca-Atlacomulco Km. 15.5, San Cayetano de Morelos, Toluca 50200, Estado de México, Mexico; saul_holic@hotmail.com; 7 Departamento de Análisis Instrumental, Universidad de Concepción, Barrio Universitario s/n, Concepción P.O. Box 160-C, Concepción 4070386, Mexico; c.mardones@udec.cl

**Keywords:** polylactic acid (PLA) film, stilbenes, food packaging, surface microbial adhesion

## Abstract

The aim of this study was evaluation of the physico-chemical properties and adhesion of microorganisms on poly(lactic acid) (PLA)-based films loaded with grapevine cane extract (5–15 wt%). The films were processed in a compression molding machine and characterized by mechanical, thermal, water vapor barrier and microbiological tests. The best physical-chemical properties for PLA film containing 10 wt% of extract were obtained. The addition of 10 wt% of extract into PLA films led to decrease of tensile strength for 52% and increase in elongation at break for 30%. The water vapor barrier of this film formulation was enhanced for 55%. All films showed thermal stability up to 300 °C. The low release of the active compounds from films negatively influenced their antimicrobial and antifungal activity. *Botrytis cinerea* growth inhibition onto PLA containing extracts (PLA-E) films was in the range between 15 and 35%. On the other side, PLA/extract films exhibited the antiadhesive properties against *Pseudomonas aeruginosa*, *Pectobacterium carotovorum*, *Saccharomyces pastorianus*, *and Listeria monocytogenes*, which could imply their potential to be used as sustainable food packaging materials for preventing microbial contamination of food.

## 1. Introduction

Plastic production reached 359 million tons worldwide in 2018, among which 40% was used in the packaging sector [1]. The use of plastic in packaging industry has been increased mainly because of the growing population, the expansion of markets, and the need to reduce food waste [2]. However, the environmental concerns about the plastic impact on the planet´s pollution have been shifting the focus toward the development of functional biodegradable packaging materials [3].

Polylactic acid (PLA) is a promising thermoplastic polymer, primarily obtained from lactic acid that is produced from sugar feedstock (corn, wheat, potato, sugarcane, cassava root). It is a renewable, processable and completely biodegradable material classified as GRAS (Generally Recognized as Safe, GRAS) for food packaging [4]. PLA is the most important commercial biodegradable polymer representing 47% of total biodegradable polymer consumption [5]. It is a material with high thermal stability, transparency and moderate barrier properties [6,7,8]. PLA-based materials show similar barrier properties to polystyrene (PS), a slow-crystallizing pattern similar to poly (ethylene terephthalate) (PET), and thermal stability to PVC [9,10]. Up to date, PLA has been widely investigated to obtain functional biodegradable packages and as a potential material in the sector of active food packaging [11,12,13,14]. It is presented in the literature that incorporation of metal oxide nanoparticles (TiO_2_, ZnO or Ag_2_O) [15,16,17,18,19,20] into the PLA matrix can improve the mechanical, barrier and antimicrobial properties of the final material. However, the use of metal oxides in food packaging materials poses severe concerns regarding health risks. Hence, there is an increased effort to identify and utilize natural antimicrobials that can provide efficient biological safety of packages and food products simultaneously.

**Figure 1:**
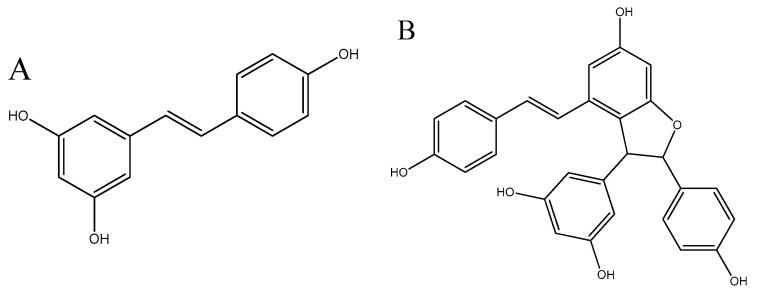
Chemical structure of trans-resveratrol (**A**) and trans-ε-viniferin (**B**) stilbenes.

In order to limit the deterioration process of food, the use of plant extracts and its by-products in food packaging has been proposed [21,22,23,24,25]. The winery industry generates waste during agricultural practices (pruning and exfoliation), vinification, and distillation processes [26]. This waste is found out to be a rich source of polyphenols with antimicrobial and antioxidant properties and it is generated yearly in high volumes [27]. Accumulation of grapevine cane is estimated between 2–5 tons/ha each year [28]. This lignocellulosic residue is generated during annual pruning and is traditionally incorporated into the soil through composting, or used as fuel [29]. This by-product is a good source of high added bioactive compounds such as phenolic acids, flavonoids and stilbenes [30]. Stilbenoids in Vitis vinifera are accumulated mainly in wood tissues and constitute a defense system against biotic and abiotic factors [31]. The main stilbene derivative products are trans-resveratrol and its dimer trans-ε-viniferin (Figure 1) and they possess antifungal and preservative properties [32]. New strategies for the valorization of grapevine canes are facilitated after the permission of resveratrol as a novel food ingredient in the European Union (Commission Implementing Decision (EU) 2016/1190), with potential use in cosmetic, food, and pharmaceutical sectors [33]. Hence, the aim of this study was evaluation of physical and biological activity of PLA/grapevine cane extract films produced by the compression molding technique. The morphological structure of films was studied by scanning electron microscopy (SEM), thermal stability by thermogravimetric analysis (TGA), mechanical analysis by tensile test, barrier properties by water vapor permeability test, and biological activity by antifungal/antimicrobial analysis toward various food pathogens and surface adhesion test of films toward food pathogens. To the best of our knowledge, grapevine cane has not been used previously in literature as a source of bioactive components to improve biological activity of PLA-based films. Up to date, only grape seed extract [34] and grape syrup [35] was studied for incorporation in PLA-matrix. Moreover, the films loaded with grapevine cane extract were reported in literature only in our previous paper, where physical-chemical properties of thermoplastic starch/grapevine cane extract materials were studied [36]. The focus of this work was to test functionality of this extract in more stable thermoplastic biopolymer, in order to extend its potential usage as bioactive compound in different biopolymer films.

## 2. Materials and Methods

### 2.1. Materials

Polylactic acid (PLA), IngeoTM grade 3251 D with M_W_ = 130,000; M_W_/M_n_ = 1.7; A ratio of isomers ~96:4; specific gravity = 1.24 g/cm^3^ was purchased from Nature Works LLC (Nebraska, MI, USA). Polyethylene glycol with a molecular weight of 1500 mol/g was obtained from Sigma Aldrich. The purity of all chemicals was 99.9%.

The grapevine cane extract was obtained from the Department of Instrumental Analysis at the University of Concepcion, Concepción, Chile. The detailed procedure to obtain an extract from grape cane residues was described in our previous paper [36]. Namely, Pinot Noir grape canes were collected from healthy plants in an organic vineyard (chemical fertilizers, pesticides, fungicides, not employed), Viña De Neira, located in Ránquil, Itata Valley, the Biobio region in South Chile (36°36′50.33″ S, 72°39′40.63″ W at 279 m of altitude). Firstly, the grapevine cane was pruned, cut in pieces of 30–50 cm stored for over 3 months at 19 °C ± 5 and 70% relative humidity. Then, the cane was chopped in a Retsch grinder (Retsch, Haan, Germany) (model SM) at 300–2000 rpm. Afterwards, it was placed in a reactor with ethanol/water solution (80:20 *v*/*v*) at 80 °C for 100 min. During exposed time solvent evaporated and the extract was collected and spray-dried using a BHS Büttner-Schilde-Haas AG dryer (Büttner, Hanover, Germany) at a rate of 15 mL/min, operated with an inlet temperature of 160 ± 5 °C, outlet temperature at 60 ± 5 °C, and injected compressed air at 40 MPa. The spray-dried grape cane extract (E) was stored under room temperature in aluminum containers.

*Botrytis cinerea* was obtained from the fungi collection in the Laboratory of Applied Microbiology and Mycology of the University of Concepcion, *Listeria monocytogenes ATCC 7644*, *Pectobacterium carotovorum ATCC 15713*, *Pseudomonas aeruginosa ATCC 10145*, and *Saccharomyces pastorianus ATCC 2345* were obtained from CIAD, Hermosillo, Sonora, Mexico. The strains were stored at −70 °C in saline solution (NaCl 0.9%) containing 20% (*v*/*v*) glycerol as a cryoprotectant.

### 2.2. Films Preparation

Masterbatches were prepared by mixing PLA with plasticizer (PEG1500) and the grapevine cane extract at different ratios (see Table 1) using a Brabender Mixer 50 EHT (Brabender, Duisburg, Germany), at 170 °C for 5 min and 60 rpm. The obtained masterbatches were grinded using a low-speed granulator (model MF10B, IKA® Werke, Staufen, Germany). Films were made in a Labtech LP-20B hydraulic press (Yannawa, Bangkok, Thailand). The grinded mixture was pre-heated for 8 min, then a pressure of 31 bar was applied for additional 3 min at 170 °C and finally the resulting films were allowed to cool down for 1 min before being demoulded. The thickness of films was checked at 10 different surface points by digital micrometer (Mitaka, Tokio, Japan) with a precision of 0.001 mm. The average thickness of all films was 0.015 ± 0.001 mm. The pure PLA and PLA/grapevine cane extract films (PLA-E) were conditioned at 50% of RH for 48 h before further characterization.

### 2.3. Films Characterization

#### 2.3.1. SEM

The surface of formulated films was observed by an ETEC autos-can SEM (Model U-1, University of Massachusetts, Worcester, MA, USA). Films were fixed in a sample holder and coated with a gold layer for 3 min using an Edwards S150 sputter coater (BOC Edwards, São Paulo, Brazil), operated at a voltage at about 1 kV.

#### 2.3.2. Mechanical Analysis

A tensile test was performed using the Universal test machine Smartens 005, equipped with a 5 kN load cell (KARG Industrie Technik, Krailling, Germany), according to the ASTMD638 (2010) standard test. The width and length of the investigated films were 5 mm and 50 mm, respectively. All the analyses were carried out at 23 ± 2 °C and 50 ± 5% RH at a crosshead rate of 2 mm/min. The reported data are the average values for four measurements. The obtained parameters of the tensile test were repeatable within ±15%.

#### 2.3.3. TGA

Thermogravimetric analyses were carried out on NETZSCH TG 209 F3 Tarsus® thermal analyzer (MB and Cia, Selb, Germany). Five mg of each sample was placed in ceramic crucibles, and the machine was heated from 30 to 500 °C at a heating rate of 10 °C/min, under a nitrogen atmosphere with a nominal gas flow rate of 30 mL/min. For each composition, the thermogravimetric tests were performed in duplicate. The obtained values were within ±3%.

#### 2.3.4. Water Vapor Permeability

The water vapor permeability (WVP) of the films was determined gravimetrically following the standard method of ASTM E96-95. The metal cups with a 6.154 cm^2^ exposed area were filled with distilled water, the sample was placed on the top of the cup and sealed, and all cups were stored in an environmental chamber set at a temperature of 25 °C and an RH of 50%. The cups were weighed every day until the equilibrium was reached and water vapor transmission rate (WVTR) values, expressed in g/(h m^2^) were determined from the linear plot of weight change vs time, following the equation:

(1) $$\mathrm{WVTR}=\frac{\Delta G}{\mathrm{txA}}$$

where ΔG was the weight change (g), t was the time during which ΔG occurred (h), A was the test area cup (m^2^), and ΔG/t was the slope in the linear plot.

Water vapor permeability (WVP) was calculated according to the following equation:

(2) $$\mathrm{WVP}=\frac{\mathrm{WVTRxL}}{\Delta p}$$

where L (m) is the thickness of the film and Δp is the water pressure difference between both sides of the film (Pa). The data were presented as the mean of three measurements for each sample and the obtained values varied within ±10%.

#### 2.3.5. Release of Grapevine Cane Extract from Films and Biological Activity of Films

The release of grapevine cane extract from PLA films into aqueous food simulant (ethanol/water 50/50 *v*/*v*) was studied. Samples of 1.0 cm × 1.0 cm size were immersed in 10 mL of food stimulant, sealed and stored at 25 °C. The quantification of bioactive compounds was performed using a C-18 core–shell column (150 mm × 4.6 mm, 2.7 μm particle size, Halo, Advance Materials Technology, Delaware, WILM, NC, USA) on a Shimadzu Nexera High Performance Liquid Chromatography (HPLC) system (Kyoto, Japan), according to the previously described methodology by Saez et al. [37]. The injection volume was 15 μL, and the mobile phases were (A) 0.1% formic acid in water and (B) acetonitrile. The gradient steps were: the mobile phase B was increased from 20% to 35% at 4 min, to 44% at 7.5 min, and to 100% at 8.5 min. Afterwards, the column was washed with acetonitrile for 2 min, and re-equilibrated by use of 20% of mobile phase B for 2 min. The column oven temperature was set at 40 °C, and the flow rate was 1.5 mL min^−1^. The diode-array detection (DAD) was set at 306 nm (SPD-M20A, Shimadzu, Kyoto, Japan), while fluorescence detection was set at 330 nm for excitation and 374 nm for emission (RF-20 AXS, Shimadzu, Kyoto, Japan). External calibration curves were made using pure standards quantified at 306 nm for resveratrol and viniferin.

The antifungal activity was determined according to Kuorwel et al. [38]. Potato dextrose agar (PDA) (Bioxon, México) was prepared and sterilized at 121 °C for 15 min, and 20 mL were placed in Petri dishes (100 mm × 15 mm). Disc of 5 mm in diameter of the *Botrytis cinerea* was placed in the center of the Petri dishes. Afterward, a piece of the film (1.0 cm × 1.0 cm) was attached to the inside cover of the Petri dish. Then petri dishes were sealed with parafilm and incubated at 25 ± 2° until control (neat PLA film) reached its maximum development. Mycelial growth was measured daily using a Vernier Caliper to evaluate the diameter reached by mycelium over time. Analyses were carried out in triplicate. The results were reported as mycelial growth inhibition index (*IM*), according to the following equation:

(3) $$IM\;\left(\%\right)=\frac{C_{C}-C_{T}}{C_{C}}$$

where *C_C_* was the control growth, and *C_T_*, the growth in the treatment.

The antibacterial activity was carried out using the Kirby-Bauer method against different strains listed in Table 2. The inoculum was prepared using the direct colony suspension method in saline solution from exponential phase cultures (18 h in Mueller-Hinton broth); each inoculum was prepared at 1 × 10^8^ CFU mL^−1^. A cotton swab was used to inoculate the appropriate agar medium with the bacterial suspension and distributed evenly over the entire surface of the Petri plates. It was left to dry for 10 min and afterward, a piece of 1 cm^2^ of each film was deposited on the surface. The inhibition zones were determined after incubation of samples for 24 h at appropriate conditions indicated in Table 2. All tests were performed in triplicate and the results did not differ more than 10%.

#### 2.3.6. Bacterial Adhesion on Polyethylene Terephthalate (PET), Polystyrene (PS), and PLA-E Surfaces

The bacterial adhesion on film surfaces was carried out according to the method reported by Vazquez-Armenta et al., [39] with some modifications. The most common commercial food packaging materials, PET and PS, were used as control samples. Firstly, PET and PS films (1.0 cm × 1.0 cm) were placed in ethanol and subjected to an ultrasonic bath for 30 min. Then they were washed with distilled water and dried at 50 °C for 30 min in an oven. PET, PS, PLA, and PLA-E (1.0 cm × 1.0 cm) films were sterilized by UV light for 2 min and under sterile conditions placed in Mueller Hinton broth test tubes. The bacterial suspension (see Table 2) was added at concentrations of 1 × 10^8^ CFU mL^−1^. The inoculum was prepared from an exponential phase culture in Mueller Hinton broth. The test tubes with films were incubated for 24 h. Subsequently, films were removed and washed with saline solution to remove non-adherent cells. Films were placed in 5 mL of saline solution and subjected to an ultrasonic bath for 5 min to release the adherent cells. From the bacterial suspension obtained after sonication, serial dilutions were carried out to determine the number of adherent bacteria per unit area as log CFU cm^−2^ by plate count on Mueller-Hinton agar after incubation for 24 h. All tests were performed in triplicate, and the results did not differ more than 10%.

#### 2.3.7. Statistical Analysis

The experimental data were subjected to an analysis of variance using the Statistical Analysis Software SPSS® v.11.0 (IBM, Chicago, IL, USA). Duncan’s new multiple range test (MRT) was used to compare the differences among means at the level of (*p* < 0.05).

## 3. Results and Discussion

### 3.1. SEM Analysis

**Figure 2:**
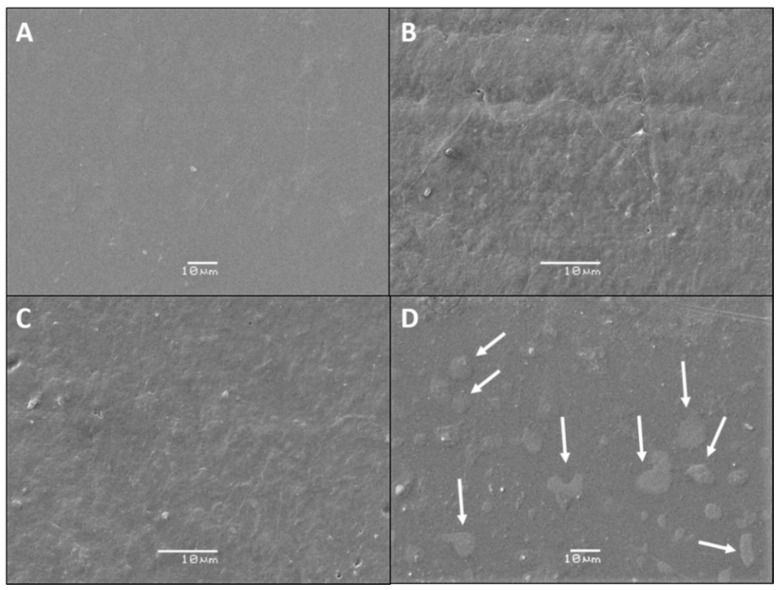
Scanning electron microscopy (SEM) micrographs of: (**A**) PLA, (**B**) PLA-E5, (**C**) PLA-E10, and (**D**) PLA-E15. White arrows indicate the presence of stilbene-rich extract agglomerates on the biopolymer film surface.

The SEM micrographs of PLA-E surface films are shown in Figure 2. An external regular and smooth surfaces are typical for PLA film [4]. The visual observation shows that all PLA films with grapevine cane extract have yellow appearance and homogenous surface. As it is shown in Figure 2, a smooth surface is obtained for PLA film, whereas films containing the grapevine cane extract show a slightly rough surface. Similar changes in the roughness were observed by Arrieta et al., [10] in uniform PLA films reinforced with yerba mate lignocellulosic residues. As the concentration of extract increases in the PLA matrix, the surface is rougher and denser. In the film PLA-E15, a random agglomeration of extract particles is visible, suggesting that 15 wt% of the extract is very high concentration for a good and homogenous dispersion into PLA matrix. A good dispersion of the extract at a lower concentration can be explained as a response to the hydrogen bonding formation between the carbonyl groups of PLA and the hydroxyl groups of resveratrol and viniferin [40]. Similar results were obtained in our previous paper, where the addition of grapevine cane extract in thermoplastic starch led to a more dense rough surface; with a higher concentration of extract in the films, the agglomeration of extract particles occurred [36].

### 3.2. Mechanical Analysis

Mechanical resistance is a key parameter for food packaging because it should maintain mechanical resistance for safe transport and food products storage. The mechanical properties of PLA films are presented in Table 3. The results show that the addition of grapevine cane extract leads to a decrease in tensile strength (TS) and Young modulus (E) in comparison to control PLA films. On the other side, the plasticity of films is enhanced by incorporating grapevine cane extract up to 10 wt% into PLA matrix. However, with a further increase of grapevine cane extract amount in film, the plasticity, tensile strength and Young modulus decreases significantly (*p* < 0.05). It is known that dispersion of additives/fillers in the polymer matrix are the main parameters which control the effective stress transfer at the interface of the matrix and the filler [41]. In case of PLA-E15 sample, lower dispersion of grapevine cane extract into PLA matrix, leads to weaker interaction between extract and PLA, resulting in decrease of mechanical strength and elongation at break. This result is in agreement with the SEM analysis described in Section 3.1. The mechanical behaviour of PLA-based films was expected, because it was confirmed in the literature that phenolic compounds in the polymeric matrix had a plasticizing effect [42]. Moreover, the addition of resveratrol into the PLA matrix was associated with a reduction in mechanical strength and an increase in the plasticity of films [43]. Agustin-Salazar et al. also obtained a reduced tensile strength when 1% *w*/*w* of resveratrol was loaded into the PLA matrix [44]. Although tensile strength is reduced within the incorporation of extract into PLA matrix, the mechanical resistance of films is still comparable with commercially used food packaging materials like LDPE (7–17 MPa) and HDPE (20–40 MPa) [45].

### 3.3. TGA

**Figure 3:**
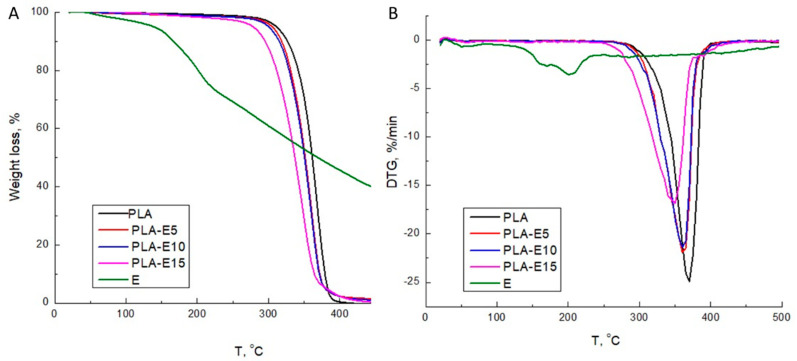
TG (**A**) and DGT (**B**) diagrams of stilbene-rich extract and PLA-based films.

The thermogravimetric analysis was carried out to evaluate the influence of the extract on the thermal decomposition of PLA-based films. As shown in Figure 3 and Table 3, PLA decomposes in a single weight loss step with an initial degradation temperature (T_max_) at 369 °C. T_onset_ and T_max_ values decrease with the extract incorporation in the PLA matrix; this effect is more pronounced for the PLA-E15 sample. The DGT curve of PLA-E15 displays a broad shoulder degradation peak, suggesting the decomposition of some bioactive components of the extract and PLA chains [36]. Ortiz-Vazquez et al. [46] and Agustin-Salazar et al. [44] also observed a decrease in the thermal stability of PLA films that contain butylated hydroxytoluene (BHT) and resveratrol, respectively. As the authors suggested, the decrease in PLA films’ thermal stability was obtained due to presence of degradation products formed during materials processing. The authors proposed that the decrease in PLA degradation temperatures in the presence of resveratrol was attributed to the phenol hydroxyl-initiated ester interchange process, which gave oligomers and low molecular weight species that volatilized at lower temperatures. Although the thermal stability of PLA/grapevine cane extracts films is slightly reduced, the T_onset_ of these films is around 300 °C, which is significantly above the processing temperature region of PLA-materials (200 °C), thus confirming that these formulations could be processed without risking high thermal degradation.

### 3.4. Water Vapor Barrier Properties

The water vapor barrier of material is a critical property to define the type of food that can be packed. The results of the water vapor properties of PLA-based films are shown in Table 3. The WVP of the control PLA film is 3.52 × 10^−11^ g/m Pa s, which is comparable with the results obtained in literature for other PLA- based materials [47]. The addition of grapevine cane extract leads to decreased water vapor permeability values of films, thus increasing the water vapor barrier. Interestingly, an increase of extract concentration up to 10 wt% in PLA films results in decrease of water vapor permeability. This outcome is probably attributed to the lipophilic nature of bioactive components in extract (resveratrol and viniferin). This fact was also observed by Hwang et al. [43], which suggested that the addition of antioxidants, such as resveratrol made films more hydrophobic and less prone to water vapor diffusion and permeability. Martins et al. (2018) reported incorporating green tea extract in the PLA matrix also decreased water permeability [22]. The same was reported by Talebi et al. [48] when essential oils (0.5–1%) were added into PLA films. WVP value of PLA/-E15 film is higher than for PLA-E5 and PLA-E10 samples but still lower than for neat PLA films, which is a result of the non-homogeneous distribution of extract at high concentration in the PLA matrix.

### 3.5. Release of Extract from PLA Films and Biological Activity

The released extract from the films was monitored at days 2 and 7 to evaluate the antimicrobial compounds’ migration. Simultaneously, the antifungal performance for all samples was also assessed. The inhibition rate of *B. cinerea* growth on the PLA-E films and neat PLA samples is shown in Table 4. A significantly (*p* < 0.05) higher stilbene-rich extracts release was observed in PLA-E15 films from 2 to 7 days. In general, a low migration level of extract from the films is achieved, which is well below the allowed overall migrations limits of substances from food packages (10 mg/dm^2^) authorized by Regulation EU No. 10/2011. On another side, the antifungal activity increases with the higher loadings of extract in the polymer matrix. The *B. cinerea* inhibition determined in PLA-E15 film was significantly higher (*p* < 0.05) than other PLA-E samples. The increased inhibition rate of *B. cinerea* is associated with the extract concentration in films and its release from the biopolymer matrix. The stilbene trans-resveratrol is a phytoalexin produced by the plant tissue in response to environmental stresses, such pathogen attack [49]. The antifungal activity of resveratrol against *B. cinerea* has already been proved [50,51]. However, the maximum inhibition growth of *B. cinerea* onto PLA films reaches only 35%. Moreover, films do not possess any antimicrobial activity toward tested pathogens: *Listeria monocytogenes*, *Saccharomyces pastorianus*, *Pseudomonas aeruginosa*, and *Pectobacterium carotovorum*.

The formation of the compact and dense structure shown in SEM analysis probably prevents the higher release of extract from the matrix, resulting in low antifungal activity. Moreover, the release of an active agent into the food simulant involves several factors, such as type of solvent and extract solubility. Hence the low chemical affinity and solubility of resveratrol and viniferin in the aqueous solvent could explain the observed effect [52]. Similar results were found in our previous paper, where the incorporation of grapevine cane extract into thermoplastic starch led to low antifungal activity toward *B. cinerea* (45% of IG) [36]. On the other hand, Pastor et al. demonstrated that addition of resveratrol into a chitosan matrix did not provide antifungal activity toward tested fungi [53].

### 3.6. Adhesion on Polyethylene Terephthalate (PET), Polystyrene (PS), and PLA-E Surfaces

**Figure 4:**
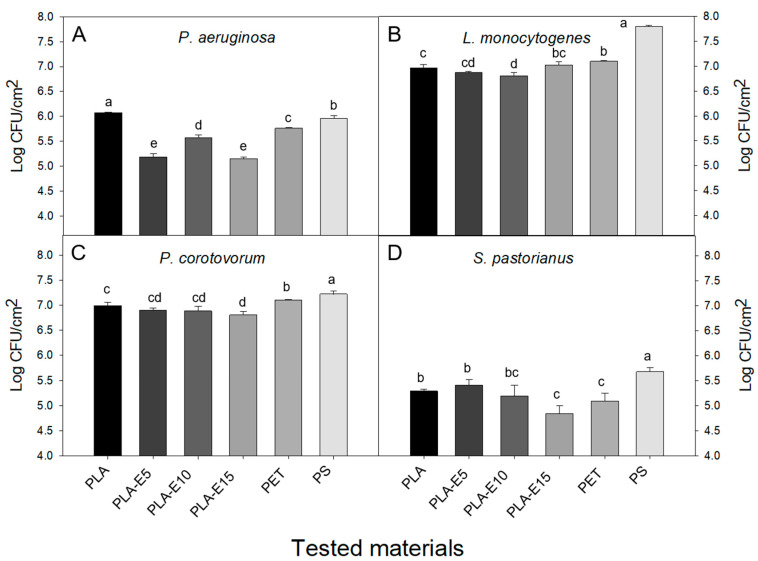
Adhesion of *P. aeruginosa* (**A**), *L. monocytogenes* (**B**), *P. corotovorum* (**C**) and *S. pastorianus* (**D**) microorganisms onto PLA, PLA-E films, polyethylene terephthalate (PET) and polystyrene (PS) samples. The bars indicate standard error (*n* = 3). Different letters indicate significant differences between CFU counts according to the Duncan test (*p* < 0.05).

Food packaging can be contaminated inside and outside. The outer food packaging surface can be contaminated during the supply chain and become a consumer potential risk due to the possible hand microbial transference in consumption moment [54]. Hence, it is important to develop biobased materials to prevent microbial proliferation on both sides of packages and minimize potential health risks. A bacterial adhesion test was performed onto PLA samples and commercially used food packaging plastics, PET and PS. The film samples were tested toward one Gram + *Listeria monocytogenes ATCC 7644*, Gram-, *Pseudomonas aeruginosa ATCC 10145*, and *Pectobacterium carotovorum ATCC 15713*, and yeast pathogen *Saccharomyces pastorianus ATCC 2345*. The attached pathogens onto PET, PS, PLA, and PLA-E surfaces are presented in Figure 4. In all cases, the order of attached pathogens on the tested surfaces was as follows: *Listeria monocytogenes* > *Pectobacterium carotovorum* > *Pseudomonas aeruginosa* > *Saccharomyces pastorianus*. PS sample shows the highest cell adhesion in three of four tested microorganisms, except in *P.* *aeruginosa*. At the same time, PET showed a significantly (*p* < 0.05) higher number of adhered cells than PLA films for *L. monocytogenes* and *P. carotovorum* strains. Conversely, it showed a significant (*p* < 0.05) higher antiadhesive effect than PLA for remaining strains (*S. pastorianus* and *Pseudomonas aeruginosa*). Kirstein et al. [55] also found diverse microbial biofilm in PLA, PET, and PS plastics in marine environments. It is well known that plastic materials provide a fixing substrate for microbial communities and that act as vectors causing severe ecological effects, such as the spread of pathogens, genes resistance, biological invasion, and production of new species [56]. It is interesting to note that the addition of grapevine cane extract into the PLA matrix leads to a reduction of cell adherence. The higher inhibition of attached cells is related to the structure and active components in PLA-E films (resveratrol and viniferin). Namely, it has been previously proved the antimicrobial efficacy of phenols from grape by-products against Gram (+) and Gram-negative (−) bacteria [57]. Additionally, Vazquez-Armenta et al. reported the anti-adherent effect of phenolic compounds of grape extracts, where the adhesion of *L. monocytogenes* onto stainless steel and polypropylene surfaces was reduced [39]. However, in other studies, it was shown that the resveratrol activity was dependent on the type of microorganism [58]. Interactions of phenolic compounds presented in extracts could show synergic, additive, or antagonistic effects [39], which resulted in less adherence of microorganisms onto PLA-E films.

The most significant anti-adhesion effect is observed toward *P. aeruginosa* (Figure 4A)*,* where the number of attached cells decreases from 5.95 (PET), 5.76 (PS), and 5.67 (PLA) to 5.15 UFC/cm^2^ (PLA-E15). The PLA-E15 result is significant (*p* < 0.05) different from all polymers. Adhesion of *Listeria monocytogenes* (Figure 4B) is reduced in a significant (*p* < 0.05) manner from 7.1 (PET), 7.80 (PS), 7.0 UFC/cm^2^ (PLA-E15) to 6.9 (PLA). In contrast, the adhesion of *Pectobacterium carotovorum* (Figure 4C) is reduced significantly (*p* < 0.05) from 7.22 (PS), 7.16 (PET), 7.05 (PLA), to 6.8 UFC/cm^2^ (PLA-E15). The adherence of *Saccharomyces pastorianus* cells (Figure 4D) was 5.09 (PET), 5.67 (PS), 5.29 (PLA), and 4.84 UFC/cm^2^ (PLA-E15), the latter being the one with a significantly (*p* < 0.05) higher anti-adherent activity.

As it can be noticed from Figure 4, PLA-E15 is the material with the best performance in preventing the adhesion of tested pathogens. The lowest concentration of attached pathogens in the PLA-E sample is related to the highest concentration of active components in the film (resveratrol and viniferin). This result is in agreement with the literature, where it was previously confirmed that polyphenolic compounds could act as a barrier for bacteria adhesion on materials’ surfaces [39]. According to results related to biological activity and adhesion tests, it can be concluded that although PLA-E films do not possess good biological activity, they have good antiadhesion properties toward tested pathogens. PLA/extract films show a similar adhesion pattern of food like commonly used food packaging material. Hence, PLA-E films can be sustainable materials for inhibition of food contamination.

## 4. Conclusions

In this study, the physico-chemical and biological properties of PLA- grapevine cane extract films have been studied, in order to obtain potential sustainable food packaging materials. The results have demonstrated that mechanical, water vapour related, and biological properties of the obtained films can be optimized in a concentration-dependent manner by incorporating grapevine cane extract into PLA-matrix. The tensile strength values of PLA/E films are in the range of 16–23 MPa, which is similar to the values for the commercially used food packaging materials like LDPE and HDPE. Moreover, the elongation at break of films increases after addition of extract for 26% (PLA-E5), 37% (PLA/E10), and 30% (PLA-E15), when compared with neat PLA film. In addition, water vapor barrier property has been improved for 50% (PLA-E5), 55% (PLA-E10), and 28% (PLA-E15). On the other side, thermal stability of all films is maintained up to 300 °C, which is significantly above processing temperature (170 °C). The PLA/E films show low antifungal activity toward *B. cinerea* (up to 35%). However, films have good antiadhesion properties toward various microorganisms and in the same order as commercially used food packaging like PS and PET. Hence, the results obtained in this work demonstrate that PLA-E films can find potential application in the food packaging sector to prevent external food contamination during transport and storage.

## Figures and Tables

**Table 1:** Formulation of the polylactic acid (PLA) based films.

Sample	PLA (wt%)	PEG1500 (wt%)	Grapevine Cane Extract (wt%)
PLA	95	5	0
PLA-E5	90	5	5
PLA-E10	85	5	10
PLA-E15	80	5	15

**Table 2:** The microbial strains and general culture conditions.

Strain	Type of Microorganism	Cultivation Temperature (°C)
*Listeria monocytogenes* ATCC 7644	Gram positive (+)	37 ± 2
*Saccharomyces pastorianus* ATCC 2345	Yeast	30
*Pseudomonas aeruginosa* ATCC 10145	Gram-negative (−)	37 ± 2
*Pectobacterium carotovorum* ATCC 15713	Gram-negative (−)	25 ± 2

**Table 3:** Mechanical, thermal, and water vapor barrier parameters of PLA and PLA-based films.

Sample	E (MPa)	TS (MPa)	e (%)	T_onset_ (°C)	T_deg_ (°C)	WVP × 10^11^ (g/m s Pa)
PLA	735 ± 17 a	33.1 ± 0.08 a	27.4 ± 1.0 c	304	369	4.74 ± 0.31
PLA-E5	512 ± 19 b	23.8 ± 1.3 b	34.5 ± 1.5 b	302	364	2.37 ± 0.12
PLA-E10	342 ± 12 c	19.6 ± 0.09 c	37.6 ± 1.2 a	297	363	2.14 ± 0.11
PLA-E15	325 ± 45 c	16.3 ± 2.1 c	35.6 ± 3.5 ab	297	351	3.40 ± 0.47

Mechanical data is presented as means ± standard error (*n* = 4). Different letters indicate significant differences between treatments according to the Duncan test (*p* < 0.05).

**Table 4:** Release of extract and inhibition of mycelial growth of *B. cinerea* onto PLA-E samples.

Sample	Release of Extract (mg/L)		Inhibition of Mycelial Growth (%)	
	2 Day	7 Day	2 Day	7 Day
PLA-E5	1.66 ± 0.12 b	1.90 ± 0.18 b	16.2 ± 2.1 c	4.9 ± 2.7 c
PLA-E10	1.73 ± 0.15 ab	2.23 ± 0.21 b	22.5 ± 1.7 b	25.0 ± 3.9 b
PLA-E15	2.03 ± 0.18 a	4.39 ± 0.46 a	34.7 ± 0.4 a	35.8 ± 1.1 a

Data are presented as means ± standard error (*n* = 3). Different letters indicate significant differences between treatments according to the Duncan test (*p* < 0.05).
